# Concurrent EGFR mutation and SMARCA4 deficiency in non-small cell lung cancer: A case report and literature review

**DOI:** 10.1097/MD.0000000000040081

**Published:** 2024-10-11

**Authors:** Weiping Dai, Taidong Li, Yujiao Li, Chaopeng Chen, Xiang Zhang, Pingan Zhou, Bin Qi

**Affiliations:** a Department of Pathology, Central Hospital of Guangdong Provincial Nongken, Zhanjiang, Guangdong Province, China; b Department of Oncological Surgery, Central Hospital of Guangdong Provincial Nongken, Zhanjiang, Guangdong Province, China.

**Keywords:** EGFR mutation, immune checkpoint inhibitors, non-small cell lung cancer, SMARCA4 deficiency

## Abstract

**Rationale:**

SMARCA4-deficient non-small cell lung cancer (NSCLC) represents a highly aggressive subtype with poor prognosis. While clinical studies have identified common co-mutations in TP53, LRP1B, STK11, KEAP1, and KRAS, actionable driver mutations such as EGFR or ALK are rarely reported in conjunction with SMARCA4 deficiency. This case presents a rare instance of NSCLC featuring both an EGFR exon 21 L858R mutation and SMARCA4 deficiency, highlighting the challenges in treatment and the need for novel therapeutic strategies.

**Patient concerns:**

A 79-year-old female patient presented with concerns of a lung mass, suspected to be peripheral lung cancer based on diagnostic imaging.

**Diagnoses:**

Histopathological evaluation confirmed SMARCA4-deficient NSCLC. Molecular genetic analysis further revealed an EGFR exon 21 L858R mutation.

**Interventions:**

The patient was initially treated with osimertinib, an EGFR tyrosine kinase inhibitor. Upon disease progression, treatment was adjusted to include anlotinib in combination with ongoing osimertinib.

**Outcomes:**

The initial treatment with osimertinib led to partial remission. However, disease progression necessitated a change in therapy. The combination treatment stabilized the disease temporarily, achieving a stable disease status.

**Lessons:**

This case underscores the transient efficacy of targeted therapy in SMARCA4-deficient NSCLC with concurrent EGFR mutations. It highlights the need for continuous therapeutic adjustments and emphasizes the importance of further research into effective strategies for treating this complex and challenging subset of NSCLC, as current modalities have limitations in sustained efficacy.

## 1. Introduction

SMARCA4-deficient non-small cell lung cancer (NSCLC) is an uncommon primary malignant epithelial tumor originating in the lung, classified as a distinct clinicopathologic entity in the World Health Organization Classification of Thoracic Tumors, published in May 2021. The gene SMARCA4 on chromosome 19q13 encodes the BRG1 protein, a component of the SWI/SNF chromatin remodeling complex, pivotal in tumorigenesis and progression.^[1]^ Representing about 8% to 11% of lung cancers, SMARCA4-deficient NSCLC exhibits high malignancy, aggressive behavior, and poor prognosis, generally demonstrating suboptimal responses to cytotoxic chemotherapy.^[2]^ Rarely do these NSCLCs harbor common oncogenic driver mutations such as EGFR, ALK, and ROS1, and no standardized treatment protocols exist for this subtype. Accordingly, we present a case of SMARCA4-deficient NSCLC with a concurrent EGFR mutation, offering clinical insights for diagnosis and management and contributing to a deeper understanding of the disease’s clinical features and therapeutic strategies. In accordance with the CARE (Case Report) guidelines, the patient has provided consent for the publication of this case.

## 2. Case presentation

### 2.1. Patient information

A 79-year-old female was evaluated for an insidious onset of right cervical and shoulder pain that commenced in October 2023. The pain, initially mild and manageable, progressively intensified but was ameliorated with oral analgesics. The patient denied experiencing hemoptysis, dyspnea, respiratory distress, chest tightness, nasal congestion, rhinorrhea, vertigo, or cephalalgia.

On November 22, 2023, a comprehensive diagnostic assessment was performed using chest computed tomography (CT), which disclosed an irregular mass approximately 59 mm × 43 mm in size in the lateral segment of the right middle lung lobe. The imaging suggested peripheral lung cancer with potential mediastinal and hilar lymph node involvement on the right side. An enhanced CT and histological biopsy were subsequently recommended. The scan also suggested the possibility of metastatic involvement at the right clavicular head and noted curvatures of the bilateral fourth and fifth anterior ribs, indicative of chronic fractures. An echocardiogram was suggested for further evaluation. A nodular shadow was observed in the right adrenal gland, presumed to be an adenoma; however, the possibility of metastatic disease could not be excluded, necessitating additional investigation.

The patient was admitted with a provisional diagnosis of “indeterminate right lung mass” for further evaluation and management. Clinical assessment revealed stable vital signs, unremarkable mental status, appetite, and sleep quality. The patient’s gastrointestinal and urinary functions were normal; no significant weight change was noted recently. Respiratory examination showed clear bilateral breath sounds without any signs of rales or pleural rub. No enlargement of the superficial lymph nodes was detected. Palpation of the right clavicular area revealed a firm mass measuring approximately 2 cm × 1.5 cm.

### 2.2. Diagnostic imaging, histopathological evaluation, and outcomes

#### 2.2.1. Initial diagnostic imaging and histopathological evaluation

**Figure 1.**
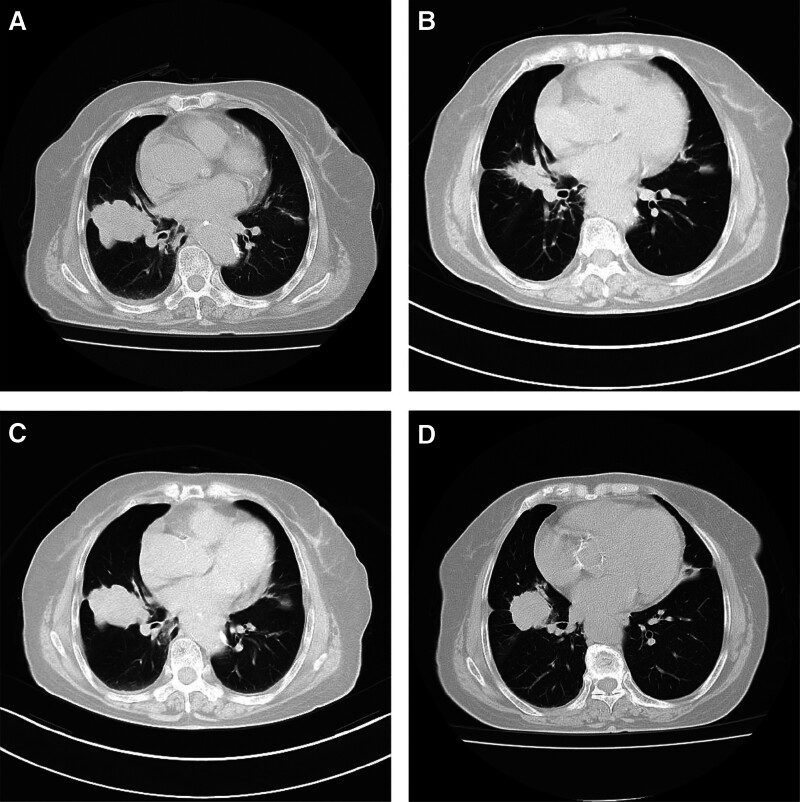
The CT imaging indicates that the boundaries of the right lung mass are indistinct, with adjacent pleural thickening and adhesion. (A) November 22, 2023: The largest cross-sectional area of the right lung mass measured approximately 59 mm × 43 mm (slice 29/3). The mass displayed a lobulated contour with heterogeneous density; no calcifications or cavitations were noted. There was invasion into the adjacent pleura, and the lesion showed marked heterogeneous enhancement on contrast-enhanced scans. (B) January 10, 2024: A follow-up CT scan of the right middle lobe showed a significant reduction in the size of the irregular mass, now measuring approximately 32 mm × 27 mm. (C) February 2024: Subsequent imaging revealed an increase in the size of the irregular mass in the right middle lobe, now measuring approximately 47 mm × 36 mm. The mass retained a lobulated appearance with heterogeneous internal density, without evidence of calcification or cavitation, and continued invasion into the adjacent pleura. Contrast-enhanced scans demonstrated pronounced heterogeneous enhancement of the tumor. (D) April 7, 2024: Compared to the findings from February 2024, there was no significant enlargement of the right lung mass.

**November 22, 2023, chest computed tomography:** An irregular mass measuring approximately 59 mm × 43 mm was identified in the lateral segment of the right middle lung lobe, suggestive of peripheral lung cancer potentially involving mediastinal and hilar lymph nodes. An enhanced scan and histologic biopsy were recommended for further evaluation. Imaging also revealed a suspected metastatic lesion at the right clavicular head and curvatures of the bilateral 4th and 5th anterior ribs, likely indicating old fractures. An evaluation using emission computed tomography was advised. Additionally, a nodular shadow in the right adrenal gland, presumed to be an adenoma, warranted the exclusion of metastasis through further abdominal CT imaging (Fig. 1).

**November 24, 2023, comprehensive chest and abdominal CT:** Confirmed the irregular mass in the right middle lobe with ongoing suspicion of mediastinal and hilar lymph node metastasis, necessitating a biopsy. The adrenal lesion remained likely adenomatous, with magnetic resonance imaging (MRI) recommended if clinically indicated. The liver and kidneys displayed multiple cysts. There was evident atherosclerosis in the abdominal aorta and its branches. Inflammatory changes were observed in the medial segment of the right middle lobe, right lower lobe, and lower lingular segment of the left upper lobe.

**Figure 2.**
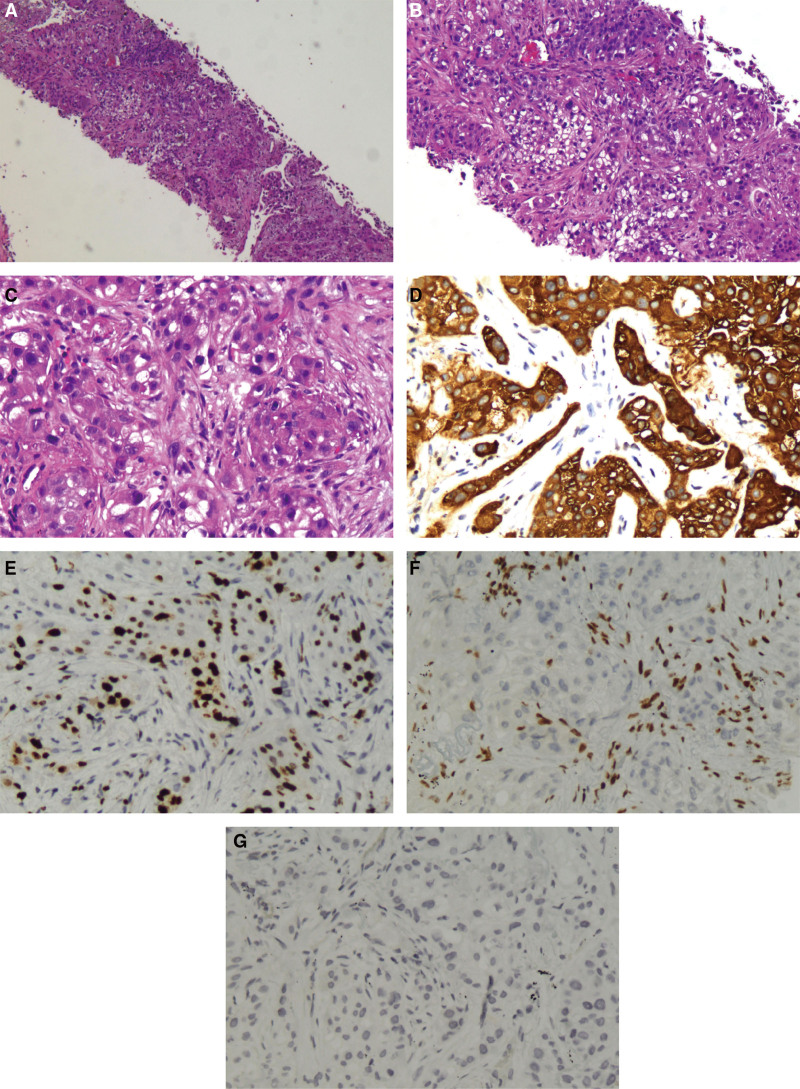
Invasive poorly differentiated adenocarcinoma, consistent with immunohistochemical findings, suggestive of SMARCA4-deficient non-small cell lung cancer. (A) Tumor cells are arranged in glandular or nested patterns of invasive growth, with nested necrosis (50× magnification); (B) tumor cells are diffusely distributed in sheet-like formations, round to epithelial-like, with vacuolated chromatin and prominent nucleoli (100× magnification); (C) the nuclei are relatively uniform, occasionally showing mild to moderate pleomorphism (200× magnification); (D) immunohistochemistry: CK7 (+); (E) immunohistochemistry: Ki-67 (60%+); (F) immunohistochemistry: SMARCA4 (−, absent); (G) immunohistochemistry: TTF-1 (−).

**December 1, 2023, histopathology report:** The specimen revealed infiltrative, poorly differentiated adenocarcinoma. Immunohistochemical staining confirmed SMARCA4-deficient non-small cell lung cancer. Immunoprofile: carcinoembryonic antigen (CEA) and creatine kinase (CK) CK7 positive; p40, Napsin A, and TTF-1 negative; c-MET weakly positive (1+); ALK-negative; p53 expressed in 30% of tumor cells; Ki-67 proliferation index at 60%; SMARCA4 absent, indicating loss; CD56 negative (Fig. 2).

**Figure 3.**
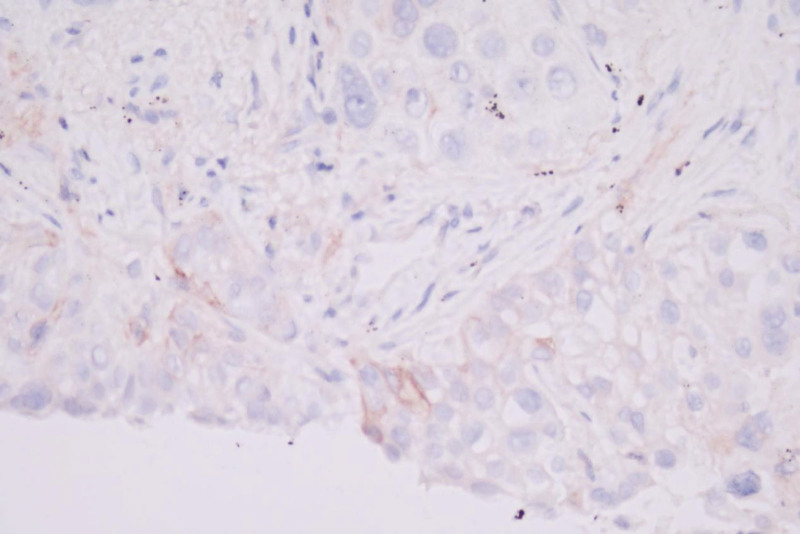
Two percent of tumor cells exhibit mild to moderate membranous expression of the PD-L1 antibody, corresponding to a Tumor Proportion Score (TPS) of 2%.

**December 4, 2023, molecular genetic analysis:** Genetic testing on a biopsy sample from November 29, 2023, showed an EGFR exon 21 L858R mutation at an allele frequency of 52.8%, 2-fold amplification of the EGFR gene, TPS 2%, and CPS 3% (Table 1, Fig. 3).

**Table 1:** Next-generation sequencing gene test results for a biopsy specimen of a right lung tumor.

Gene	Test result	Abundance	Clinical significance level
AKT1	Not detected	–	–
ALK	Not detected	–	–
ARAF	Not detected	–	–
ATM	Not detected	–	–
BARD1	Not detected	–	–
BCL2L11	Not detected	–	–
BRAF	Not detected	–	–
BRCA1	Not detected	–	–
BRCA2	Not detected	–	–
BRIP1	Not detected	–	–
CCND1	Not detected	–	–
CCND2	Not detected	–	–
CCNE1	Not detected	–	–
CD274	Not detected	–	–
CDK12	Not detected	–	–
CDK4	Not detected	–	–
CDK6	Not detected	–	–
CHEK1	Not detected	–	–
CHEK2	Not detected	–	–
CTNNB1	Not detected	–	–
DDR2	Not detected	–	–
DPYD	Not detected	–	–
EGFR	NM_005228..5 (EGFR):c.2573T > G(p.Leu858Arg)	52.8%	I
		2.0-fold	II
ERBB2	Not detected	–	–
ERBB3	Not detected	–	–
ERBB4	Not detected	–	–
FGF19	Not detected	–	–
FGF3	Not detected	–	–
FGFR1	Not detected	–	–
FGFR2	Not detected	–	–
FGFR3	Not detected	–	–
FLT3	Not detected	–	–
HRAS	Not detected	–	–
IDH1	Not detected	–	–
IDH2	Not detected	–	–
JAK1	Not detected	–	–
JAK2	Not detected	–	–
KIT	Not detected	–	–
KRAS	Not detected	–	–
MAP2K1	Not detected	–	–
MDM2	Not detected	–	–
MDM4	Not detected	–	–
MET	Not detected	–	–
MSH2	Not detected	–	–
MTOR	Not detected	–	–
MYC	Not detected	–	–
NF1	Not detected	–	–
NFE2L2	Not detected	–	–
NRAS	Not detected	–	–
NRG1	Not detected	–	–
NTRK1	Not detected	–	–

#### 2.2.2. Treatment and follow-up assessments

**January 10, 2024, chest computed tomography:** A subsequent chest CT 1 month later demonstrated a reduction in the dimensions of the mass in the right middle lobe (approximately 32 mm × 27 mm) and decreased involvement of the mediastinal and right hilar lymph nodes. This response was classified as partial remission (PR) (Fig. 1).

**February 2024, repeated chest CT and MRI:** The patient reported worsening neck and shoulder pain. Repeated chest CT scans revealed an enlarged mass in the right middle lobe, increased size of the right hilar lymph nodes, and a partial decrease in the mediastinal lymph nodes. There was significant progression in osteolytic destruction of the right clavicle, accompanied by a pathological fracture. Cranial MRI showed a reduction in the size of the metastatic lesion in the right parietal lobe. Despite ongoing targeted therapy, the disease demonstrated marked progression (Fig. 1).

**April 2024, follow-up assessment:** The disease was temporarily stabilized, achieving a stable disease status. Subsequently, the patient opted to discontinue further treatment and requested discharge (Fig. 1).

#### 2.2.3. Summary of outcomes

The patient was initially treated with osimertinib, resulting in partial remission. However, disease progression occurred within 3 months, necessitating a treatment adjustment to include anlotinib combined with ongoing osimertinib, which led to temporary disease stabilization. This case underscores the transient efficacy of initial targeted therapy and the necessity for ongoing adjustments in therapeutic strategies for SMARCA4-deficient NSCLC with concurrent EGFR mutations.

### 2.3. Diagnostic assessment

SMARCA4-deficient non-small cell lung cancer: Infiltrative poorly differentiated adenocarcinoma of the right lung with EGFR exon 21 L858R mutation, classified as stage IVB (cT3N2M1c) involving the right parietal lobe, right clavicle, and right sacroiliac joint.

### 2.4. Ethics statement

This study was approved by the Ethics Committee of the Central Hospital of Guangdong Provincial Nongken, Zhanjiang (Approval Number: [20240423]).Written informed consent was obtained from the patient’s family for the publication of this case report and any accompanying images.

### 2.5. Hospitalization and subsequent evaluations

#### 2.5.1. Laboratory and imaging assessments

The patient underwent a comprehensive diagnostic evaluation upon hospitalization. Urinalysis detected the presence of vitamin C (1+, increased) and bacteria (757.55 [particles/μL], elevated). Tumor marker analysis revealed increased levels: CEA at 17.96 ng/mL, cancer antigen 125 at 92.64 U/mL, and cancer antigen 15-3 (CA15-3) at 46.99 U/mL. The biochemical profile showed elevated CK at 184.0 U/L and total cholesterol at 6.06 mmol/L. Routine hematological parameters and fecal tests were within normal limits. An electrocardiogram displayed sinus rhythm with left axis deviation. Echocardiographic findings indicated cardiac alterations secondary to hypertension, aortic valve calcification, mild regurgitation at the aortic and mitral valves, normal systolic function of the left ventricle, and impaired diastolic function. Additional findings included hepatic and renal cysts; no abnormalities were noted in the spleen, pancreatic head, body, right kidney, ureters, or uterus on color Doppler ultrasound.

#### 2.5.2. Initial therapy and follow-up

The patient initiated treatment with osimertinib (80 mg daily) in November 2023. One month later, a chest CT scan showed a reduction in the mass size in the right middle lobe from 59 mm × 43 mm to approximately 32 mm × 27 mm. Additionally, there was a decrease in the involvement of the mediastinal and right hilar lymph nodes. This response was classified as partial remission.

#### 2.5.3. Clinical progression and treatment adjustment

By February 2024, the patient reported worsening neck and shoulder pain. A repeated chest CT scan revealed an enlargement of the mass in the right middle lobe to 47 mm × 36 mm and an increase in the size of the right hilar lymph nodes, with partial regression in the mediastinal lymph nodes. The osteolytic destruction of the right clavicle had significantly progressed, leading to a pathological fracture. Cranial MRI indicated a reduction in the size of the metastatic lesion in the right parietal lobe. Despite ongoing osimertinib treatment, disease progression was evident.

**Figure 4.**
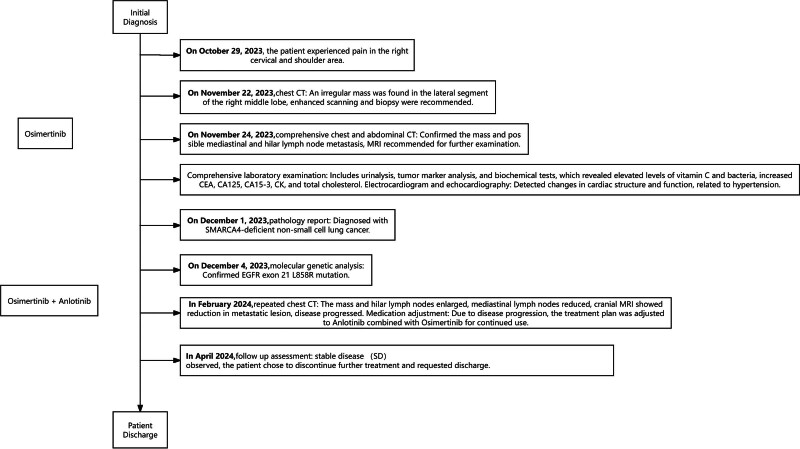
Timeline of the clinical course of the patient.

Given the progression, the patient declined chemotherapy and the treatment regimen was adjusted to include anlotinib (10 mg daily for 14 days in a 21-day cycle) combined with ongoing osimertinib. After 2 months, the disease was assessed as stable disease. The patient then chose to discontinue treatment and requested discharge (Fig. 4).

### 2.6. Literature review

The comprehensive literature search was conducted using the keywords “SMARCA4,” “EGFR,” and “ALK” across PubMed and Web of Science databases. To date, there are only 2 globally documented instances of non-small cell lung cancer (NSCLC) with concurrent SMARCA4 deficiency and mutations in EGFR or ALK (Table 2).

**Table 2:** Comparison the effect of various treatments on SMARCA4-dNSCLC with EGFR/ALK mutation.

Reference	Age/gender	Smoking	Gene	Therapy	Outcome
Sun L, et al^[3]^	60/female	Unknown	EGFR L858R	Osimertinib, 80 mg, once daily	Unknown
Sheng J, et al^[4]^	34/female	Nonsmoker	EML4-ALK	Alectinib 600 mg, twice daily	CR

CR = complete response.

The initial case pertains to a 60-year-old female with an EGFR exon 21 L858R mutation, diagnosed after she had previously undergone breast-conserving surgery over 3 years earlier, followed by postoperative radiotherapy for bilateral breast cancer. The radiotherapy regimen consisted of 5-week cycles, delivering 2 Gy per session, 5 days a week, totaling 50 Gy; additionally, a boost of 10 Gy was administered directly to the tumor bed in 5 fractions. Post-diagnosis, she commenced treatment with osimertinib (80 mg daily) and was reported to be in good health at the time of the case report, though follow-up data were lacking to provide an outcome update.

The second case describes a 34-year-old female diagnosed with a SMARCA4-deficient undifferentiated tumor that exhibited a fusion mutation involving EML4 exon 13 and ALK exon 20. Initial treatment with alectinib (600 mg, administered twice daily) led to partial remission within 6 weeks. Subsequent computed tomography scans, performed 9 months later, confirmed a significant therapeutic response, achieving complete remission. The patient experienced no adverse effects during treatment, and follow-up evaluations revealed no hematopoietic suppression or hepatic dysfunction.

## 3. Discussion

Concurrent EGFR mutation and SMARCA4 deficiency in NSCLC present a complex scenario in lung cancer pathogenesis. SMARCA4 mutations are recurrent in NSCLC, particularly in adenocarcinomas, with a prevalence of approximately 5%.^[3]^ These mutations are associated with aggressive clinical behavior.^[4]^ In NSCLC, SMARCA4 alterations have been identified in 8% of patients, highlighting their significance in this cancer type.^[2]^ SMARCA4/BRG1 loss characterizes a subset of NSCLCs lacking alterations in EGFR, ALK, and ROS1 genes.^[5]^ The loss of SMARCA4 expression is observed in up to 10% of NSCLC cases, indicating its relevance in a subset of lung cancers.^[6]^ SMARCA4-deficient NSCLC has been recognized as a distinct subtype, with unique characteristics such as neuroendocrine marker expression.^[7]^ Additionally, SMARCA4 loss in NSCLC has been associated with increased sensitivity to CDK4/6 inhibitors, suggesting potential therapeutic implications.^[8]^ Studies have shown that SMARCA4-deficient NSCLCs may have limited efficacy of immune checkpoint inhibitors due to an immune-desert tumor microenvironment.^[9]^ However, there are reports of improved performance status in SMARCA4-deficient NSCLC patients treated with immune checkpoint inhibitors.^[10]^ Furthermore, SMARCA4-deficient NSCLCs have been linked to replicative stress and ATR dependency, indicating potential vulnerabilities that could be targeted for therapeutic benefit.^[11]^

SMARCA4-deficient non-small cell lung cancer predominantly affects male smokers aged 42 to 93 years and is characterized by high metastatic potential and poor prognosis. This subtype typically resists platinum-based double-agent chemotherapy, and no targeted therapeutic options are currently available.^[12,13]^ Clinically, the disease often manifests with symptoms such as cough and sputum production and demonstrates aggressive biological behavior predominantly localized to the lungs, with frequent involvement of the pleura and vasculature.^[14,15]^ Tumor marker analysis often reveals elevated levels of CYFRA21-1 and CEA, while standard oncogenic drivers such as EGFR, ALK, and ROS1 are typically absent.^[15]^ In the present case, the patient, a nonsmoking female, was diagnosed with SMARCA4-deficient NSCLC via pathologic immunohistochemistry. Next-generation sequencing identified a concurrent mutation in EGFR exon 21 L858R, a profile not previously described in the literature.

SMARCA4-deficient neoplasms are a heterogeneous group of tumors unified by the absence of SMARCA4 expression, which can occur in various tissues. The diagnosis of SMARCA4-deficient NSCLC is predominantly based on the loss of SMARCA4 (BRG1) protein expression. Typically, genetic sequencing and protein expression assessments are employed complementarily in the diagnostic process, though the absence of SMARCA4 protein does not invariably align with genetic alterations, potentially reflecting the constraints of genomic sequencing technologies. Prior studies have classified mutations associated with SMARCA4-deficient NSCLC into 2 categories: Type 1 mutations, encompassing truncating mutations, homozygous deletions, or fusion mutations, and Type 2 mutations, characterized by missense mutations and non-truncating alterations with preserved protein expression. Moreover, prognostic discrepancies are noted between patients with preserved SMARCA4 protein expression who test positive for SMARCA4 mutations, with Type 1 mutations associated with significantly shorter survival.^[2]^ In this patient, histopathological hematoxylin and eosin (HE) staining demonstrated loss of SMARCA4 expression; regretfully, genetic analysis for SMARCA4 mutations was not conducted.

Recent clinical research increasingly focuses on the clinical characteristics, molecular phenotypes, and therapeutic approaches of SMARCA4-deficient thoracic tumors. Some case studies suggest that SMARCA4-deficient NSCLC may exhibit sensitivity to platinum-based chemotherapy, particularly in patients with low BRG1 expression. This sensitivity may be linked to DNA repair defects associated with BRG1 knockdown in NSCLC, suggesting future therapeutic strategies could explore targeting DNA repair mechanisms in conjunction with platinum-based chemotherapy.^[16]^ Additionally, some studies have noted a negative correlation between SMARCA4 expression loss and CD8 + T-cell infiltration in SMARCA4-deficient tumors, indicating a close relationship between the SMARCA4 gene and tumor immunity.^[17]^ Research by Yoan Velut et al observed that compared to patients with non-deficient SMARCA4 NSCLC, those with SMARCA4-deficient NSCLC exhibit an immunosuppressive microenvironment characterized by increased densities of FOXP3 + cells and neutrophils, but not CD8 + T cells, potentially explaining the poor prognosis, immunosuppression, and limited efficacy of PD-1 treatments in these patients.^[18]^ A retrospective study by the National Cancer Center of Japan showed that treatment with immune checkpoint inhibitors appears more promising in SMARCA4-deficient thoracic tumors, though further large-scale studies are necessary to confirm these findings.^[19]^ A study conducted in China demonstrated that compared to chemotherapy alone, immunotherapy combined with chemotherapy in first-line treatment of patients with SMARCA4-deficient tumors (including undifferentiated thoracic tumors and NSCLC) significantly extends progression-free survival, improves objective response rates, and enhances overall survival in patients who receive first-line immunotherapy compared to those treated with immunotherapy later or not at all. This suggests that patients with SMARCA4-deficient tumors should receive immunotherapy-inclusive regimens as early as possible.^[20]^

Comprehensive genomic profiling in SMARCA4-deficient NSCLC commonly identifies mutations in critical oncogenes such as TP53 (80%), LRP1B (40%), STK11 (27%), KEAP1 (27%), and KRAS (20%). Despite these insights, therapeutic options targeting these mutations remain elusive, and patients with SMARCA4-deficient NSCLC typically exhibit poorer prognostic outcomes and shorter overall survival compared to their wild-type counterparts. Importantly, immunotherapy in conjunction with platinum-based chemotherapy has demonstrated enhanced survival benefits in patients with advanced-stage disease.^[21]^ Studies conducted at the Dana-Farber Cancer Institute reveal a marked disparity in survival outcomes among SMARCA4-deficient NSCLC patients: those with concurrent KRAS mutations experience significantly reduced median progression-free survival (1.4 months vs 4.1 months) and overall survival (3.0 months vs 15.1 months) compared to KRAS wild-type counterparts.^[1]^ Although driver mutations such as EGFR, ALK, and ROS1 are infrequently associated with SMARCA4-deficient NSCLC, isolated reports suggest that patients, particularly nonsmoking females with ALK rearrangements, achieve profound therapeutic responses to first-line alectinib, highlighting the critical role of molecular diagnostics in optimizing therapeutic strategies.^[22]^ In the current case, the patient, harboring an unreported combination of SMARCA4 deficiency and an EGFR exon 21 L858R mutation, initially responded to osimertinib with partial remission. However, the progression-free survival was limited to 3 months, with subsequent rapid progression despite combination therapy with nilotinib and osimertinib. Recent investigations have elucidated that alterations in chromatin accessibility mediated by the mammalian SWI/SNF complex may facilitate osimertinib resistance in EGFR-mutant lung cancers. Moreover, the ablation of SMARCA4 is implicated in mitigating the proliferation of osimertinib-resistant neoplasms,^[23]^ providing a plausible explanation for the observed therapeutic challenges in this patient.

Preclinical studies have demonstrated that BRG1 inactivation increases tumor invasiveness and enhances sensitivity to targeted agents inhibiting oxidative phosphorylation and drugs targeting EZH2, AURKA, ATR, CDK4, and CDK6. The ongoing development of pharmacologic agents such as the EZH2 inhibitor (tazemetostat), the oxidative phosphorylation inhibitor (IACS-01759), and CDK4/6 inhibitors (palbociclib) may offer promising therapeutic options for patients with SMARCA4-deficient NSCLC, potentially improving clinical outcomes.^[13,24–26]^

This study has several notable limitations. Firstly, as a single case report of a patient with SMARCA4-deficient non-small cell lung cancer (NSCLC) with concurrent EGFR exon 21 L858R mutation, the findings may not be broadly generalizable. The relatively short follow-up period restricts the ability to evaluate the long-term efficacy and outcomes of the treatment regimen. Additionally, the patient’s decision to decline chemotherapy may have influenced disease progression and outcomes, introducing variability that could impact the reproducibility of results across a broader patient population. The literature review revealed only 2 other documented cases of NSCLC with concurrent SMARCA4 deficiency and EGFR or ALK mutations, complicating the ability to draw definitive conclusions regarding optimal treatment strategies for this specific subgroup. Furthermore, the observed heterogeneity in treatment responses among similar cases underscores the challenges in identifying universally practical therapeutic approaches for this complex disease.

## 4. Conclusion

In conclusion, the interplay between EGFR mutation and SMARCA4 deficiency in NSCLC underscores the complexity of lung cancer biology. Understanding the molecular mechanisms and clinical implications of these alterations is crucial for developing targeted therapies and improving patient outcomes. SMARCA4-deficient NSCLC represents a rare entity with distinct clinical manifestations that merit increased attention from oncologists. Current evidence predominantly indicates that immunotherapy combined with chemotherapy constitutes the most effective treatment option for advanced-stage SMARCA4-deficient NSCLC. We have reported a case of SMARCA4-deficient NSCLC with a concurrent EGFR exon 21 L858R mutation, which exhibited a suboptimal response to osimertinib treatment. However, the efficacy of targeted therapy in SMARCA4-deficient NSCLC harboring actionable driver mutations such as EGFR or ALK requires further exploration through large-scale clinical studies. Developing novel therapeutic agents may offer more promising outcomes for patients with this subtype.

## Acknowledgments

We would like to thank Lord Dr Zhibin Xu, Ph.D., from the The First Affiliated Hospital of Guangzhou Medical University for assisting with the preparation and English revision of this manuscript.

## Author contributions

**Conceptualization:** Weiping Dai.

**Data curation:** Chaopeng Chen.

**Formal analysis:** Chaopeng Chen.

**Investigation:** Taidong Li, Xiang Zhang.

**Methodology:** Xiang Zhang.

**Resources:** Pingan Zhou.

**Software:** Taidong Li, Pingan Zhou.

**Supervision:** Yujiao Li, Bin Qi.

**Validation:** Bin Qi.

**Writing – original draft:** Weiping Dai.

**Writing – review & editing:** Yujiao Li.
